# The efficacy of sodium azulene sulfonate L-glutamine for managing chemotherapy-induced oral mucositis in cancer patients: a prospective comparative study

**DOI:** 10.1186/s40780-018-0114-2

**Published:** 2018-08-13

**Authors:** Satoru Nihei, Junya Sato, Hideaki Komatsu, Kazushige Ishida, Toshimoto Kimura, Takashi Tomita, Kenzo Kudo

**Affiliations:** 1 Department of Pharmacy, Iwate Medical University Hospital, 19-1 Uchimaru, Morioka-shi, Iwate 020-8505 Japan; 2 Department of Clinical Pharmaceutics, School of Pharmacy, Iwate Medical University, 2-1-1 Nishitokuta, Yahaba-cho, Shiwa-gun, Iwate 028-3694 Japan; 3 Department of Pharmacy, Shizuoka Cancer Center, 1007, Shimo-nagakubo, Sunto-gun, Shizuoka 411-8777 Japan; 4 Department of Surgery, School of Medicine, Iwate Medical University, 19-1 Uchimaru, Morioka-shi, Iwate 020-8505 Japan

**Keywords:** Sodium azulene sulfonate L-glutamine (GA), Oral mucositis, Fluorinated pyrimidine anticancer drugs, Chemotherapy

## Abstract

**Background:**

The efficacy of sodium azulene sulfonate L-glutamine (GA) in treating oral mucositis caused by the administration of anticancer agents has not been previously elucidated. Therefore, this prospective comparative study was conducted to evaluate the efficacy of GA in treating oral mucositis caused by chemotherapy regimens involving fluorinated pyrimidine anticancer drugs.

**Methods:**

The subjects of this study were patients with oral mucositis of grade 2 or higher while on outpatient chemotherapy regimens involving fluorinated pyrimidine anticancer drugs for colorectal or breast cancer. The subjects were randomly divided into a group that received GA (the GA group) or a group that did not receive GA (the control group) by using the closed-envelope method. GA was administered three times a day every day from the first day of the regimen until the final day. The primary endpoint was the development of oral mucositis of grade 2 or higher. The secondary endpoint was the severity of oral pain, which was judged using an 11-stage numerical rating scale (NRS) ranging from 0 to 10.

**Results:**

The proportion of patients with oral mucositis of grade 2 or higher was 32.4% in the GA group and 57.6% in the control group. The GA group had a significantly lower frequency of occurrence. The changes in the NRS scores before and after the trial began were − 2.9 ± 0.6 in the GA group and − 1.2 ± 0.5 in the control group. The NRS score decreased more significantly in the GA group than in the control group (*P* = 0.046). One patient stopped GA treatment voluntarily due to nausea; other than nausea, no GA-related side effects were observed.

**Conclusions:**

GA protects against oral mucositis and reduces the severity of prevailing oral mucositis symptoms. Our findings indicate that GA is a highly safe and convenient drug.

## Background

Oral mucositis is induced by the administration of cytotoxic anticancer agents in 30–40% of patients treated with a normal chemotherapy regimen (i.e., not high-dose chemotherapy or combined radiation therapy) [1]. The risk of oral mucositis differs depending on the treatment regimen, but it is clearly exacerbated in regimens involving fluorinated pyrimidine anticancer drugs [2, 3]. The underlying mechanism of oral mucositis includes direct induction of cell death in the oral mucosa owing to the cytotoxic anticancer drug and indirect production of reactive oxygen species and inflammatory mediators in response to the administration of the cytotoxic anticancer agent, which induces epithelial cell death. Ulcers develop because of these direct and indirect effects [4]. Aggravation of oral mucositis is accompanied by pain, which can lead to difficulties in ingesting food; hence, many affected patients experience severe physical and mental distress. Thus, aggravation of oral mucositis may necessitate a decrease in the dose of the anticancer drugs involved, or halting/postponing the administration of the anticancer drug. When resorting to these measures, treatment efficacy may not be able to be maintained [5, 6]. L-glutamine, which is synthesized in muscles, is used in mucosal tissue repair [7]. L-glutamine in muscles is released into the blood and mobilized to the site of inflammation in the mucosal tissue in vivo [8]. On the other hand, patients who experience metabolic stress because of the administration of anticancer agents develop L-glutamine deficiency, which may further promote injury to mucosal tissues [9]. L-glutamine protects mucosal tissues, in addition to its role in promoting mucosal tissue repair [10, 11]. Therefore, sodium azulene sulfonate L-glutamine (GA), which is covered by health insurance, is used as an L-glutamine-containing pharmaceutical product for patients with stomach/duodenal ulcers [12]. However, the effectiveness of GA against oral mucositis resulting from oral administration of anticancer drugs has not yet been elucidated. Therefore, we conducted a prospective comparative study between patients who were administered GA and those who were not, to elucidate the safety and efficacy of GA in the treatment of oral mucositis due to a treatment regimen involving fluorinated pyrimidine anticancer agents.

## Methods

### Subjects

The subjects in this study were patients with oral mucositis of grade 2 or higher caused by an outpatient chemotherapy regimen involving fluorinated pyrimidine anticancer agents for the treatment of colorectal or breast cancer over the course of 29 months from August 1, 2014 until December 31, 2016, at Iwate Medical University Hospital. However, patients with oral side effects, liver failure, or kidney failure, in addition to those judged to have poor compliance with oral drug administration were omitted from the study. The patients were randomly assigned to two groups by using the closed-envelope method: a GA group, which received GA orally, and a control group, which did not receive GA. The experimental methods were approved by the Iwate Medical University School of Medicine Ethics Committee, and all work was conducted in accordance with the Declaration of Helsinki and ethical principles for clinical research. Written informed consent was obtained from all patients.

### Drug used to treat oral mucositis

The patients were directed to suspend 3 g of GA in water for each administration of the therapeutic agent, and to keep the suspension in their mouth for 20–50 s before swallowing. GA was used three times per day, every day from the first day of the regimen cycle until the final day. Additionally, residual GA was collected from the patients’ mouth at their next hospital visit without administering any GA to determine the state of self-administration. The use of pharmaceuticals other than GA to treat oral mucositis was permitted during the trial period; however, the use of corticosteroid hormone drugs was prohibited. Both groups were instructed on the usual oral hygiene care, which included a combination of brushing, flossing, and mouth rinsing. Patients in the control group received only usual oral hygiene care and were allowed to use mouthwash (benzethonium chloride or sodium azulene sulfonate).

### Items for evaluation

The primary endpoint was the occurrence of oral mucositis of grade 2 or higher, which was evaluated based on the Japan Clinical Oncology Group edition of the National Cancer Institute-Common Terminology Criteria for Adverse Events (NCI-CTCAE) ver. 4.0. The secondary endpoint was the intensity of oral pain, which was evaluated using an 11-stage numerical rating scale (NRS) ranging from 0 to 10. With regard to the grade of oral mucositis and the oral pain score, the symptoms at the most aggravated point during the regimen cycle were evaluated by interviewing the patients or by using a questionnaire. Furthermore, the grade of oral mucositis and oral pain scores for the cycle before the trial began were compared to those after the trial began. Additionally, changes in NRS scores were determined by comparing the NRS scores after the trial began with the NRS scores before the start of the trial. Changes in NRS scores were defined as clinically meaningful (≥30%) and highly meaningful (≥50%) based on reduction of pain intensity from baseline. The following adverse events were noted: haematotoxicity (neutropenia, decreased haemoglobin level, thrombopenia), non-haematotoxic adverse effects (nausea, vomiting, diarrhoea), and decreased liver function (increase in aspartate transaminase and alanine transaminase levels).

### Analysis methods

Each of the items evaluated was compared between the GA group and control group for analysis. The occurrence of oral mucositis, clinically meaningful and highly meaningful were analysed using a chi-square test. Changes in the NRS score for oral pain were analysed by Student’s *t*-test. The significance level was 5%, and all tests were two-sided.

## Results

### Subjects

The backgrounds of patients in the GA group and the control group are shown in Table 1. Sixty-seven patients from whom consent was acquired were divided into the GA group (34 patients) and the control group (33 patients). There were no significant differences in age, sex, tumour type, blood/biochemical test findings, chemotherapy regimen, molecular targeted drug, mouthwash, concomitant drug, and nutritional supplements between the two groups. Furthermore, the intergroup difference in NRS scores for oral pain before the trial started was not significant (GA group: 6.1 ± 2.1, control group: 5.7 ± 1.3). The baseline NRS score for all patients was NRS 4 or higher.

**Table 1:** Patients’ background characteristics

	GA group	Control group	*P* value
	(*n* = 34)	(*n* = 33)	
Age (years)^a^	62.3 ± 12.7	59.8 ± 9.4	0.396
Sex, *n* (%)			
Male	10 (29.4)	8 (24.2)	0.759
Female	24 (70.6)	25 (75.8)	
BMI, kg/m2^a^	22.6 ± 3.2	23.6 ± 2.5	0.189
ECOG PS, *n* (%)			
0	24 (70.6)	24 (72.7)	0.846
1	10 (29.4)	9 (27.3)	
Tumor type, *n* (%)			
Colorectal cancer	17 (50.0)	17 (51.5)	0.589
Breast cancer	17 (50.0)	16 (48.5)	
Tumor stage, *n* (%)			
II to III	17 (50.0)	15 (45.5)	0.710
IV	17 (50.0)	18 (54.5)	
Blood/biochemical tests^a^			
Total protein, g/dL	6.50 ± 0.45	6.40 ± 0.45	0.434
Albumin, g/dL	3.96 ± 0.37	3.84 ± 0.31	0.251
White blood cell count, × 103/μL White blood cell count, ×103/μL	4.95 ± 1.48	5.33 ± 1.67	0.383
Neutrophil count, × 103/μL Neutrophil count, × 103/μL	3.11 ± 1.28	3.21 ± 1.26	0.777
Hemoglobin, g/dL	11.9 ± 1.37	11.9 ± 1.29	0.944
Serum creatinine, mg/dL	0.66 ± 0.16	0.65 ± 0.20	0.931
Aspartate transaminase	28.3 ± 23.1	27.1 ± 23.3	0.850
Alanine transaminase	28.4 ± 25.7	26.3 ± 16.6	0.731
C-reactive protein	0.48 ± 0.68	0.34 ± 0.49	0.406
Chemotherapy regimen, *n* (%)			
5-FU + l-LV + CPT-11	7 (20.6)	8 (24.2)	0.781
5-FU + l-LV + L-OHP	10 (29.4)	9 (27.3)	
5-FU + EPI + CPA	17 (50.0)	16 (48.5)	
Molecular targeted drug, *n* (%)			
Cetuximab	1 (2.9)	1 (3.0)	0.551
Panitumumab	4 (11.8)	6 (18.2)	
Bevacizumab	10 (29.4)	11 (33.3)	
None	19 (55.9)	15 (45.5)	
Mouthwash, *n* (%)			
Sodium azulene sulfonate	1 (2.9)	3 (9.1)	0.356
Benzethonium chloride	17 (50.0)	16 (48.5)	0.901
Concomitant drug, *n* (%)			
Polaprezinc	18 (52.9)	24 (72.7)	0.094
Rebamipide	2 (5.9)	1 (4.5)	1.000
Non-steroidal antipyretic analgesic	6 (17.6)	6 (27.3)	0.508
Opioid analgesic	7 (20.6)	1 (4.5)	0.130
Nutritional supplement, *n* (%)	3 (8.8)	1 (3.0)	0.614
Numerical Rating Scale score before the trial	6.12 ± 2.07	5.68 ± 1.32	0.385

^a^mean ± standard deviation, *BMI* body mass index, *ECOG PS* Eastern Cooperative Oncology Group performance status, *5-FU* 5-fluorouracil, *l-LV* l-leucovorin, *CPT-11* irinotecan, *L-OHP* oxaliplatin, *EPI* epirubicin, *CPA* cyclophosphamide, *NRS* numerical rating scale

### Occurrence of oral mucositis

**Fig. 1:**
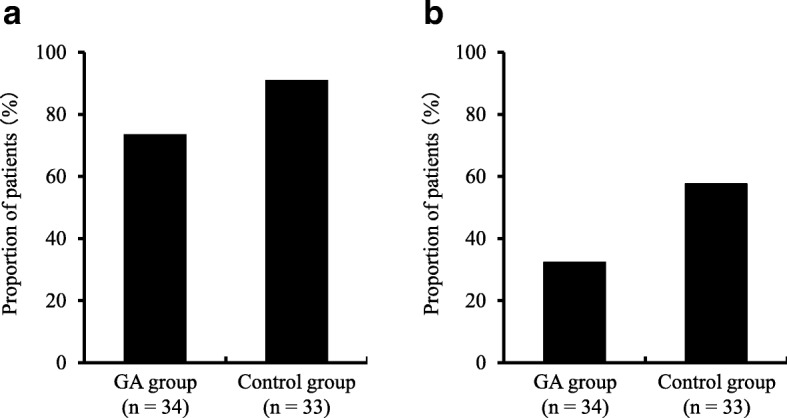
Occurrence of oral mucositis of (**a**) all grades and (**b**) grade ≥ 2. There was no statistically significant difference in the occurrence of all grades of oral mucositis between the sodium azulene sulfonate L-glutamine (GA) and control groups (**a**, *P* = 0.068, chi-square test). However, the occurrence of grade ≥ 2 oral mucositis was significantly lower in the GA group than in the control group (**b**, *P* = 0.038, chi-square test). This figure shows the proportion of patients (%) with oral mucositis

The frequency of oral mucositis in the two groups after the start of the trial is shown in Fig. 1. The proportion of patients with oral mucositis was 73.5% in the GA group (*n* = 25) and 90.9% in the control group (*n* = 30; *P* = 0.068). The proportion of patients with oral mucositis of grade 2 or higher was 32.4% (*n* = 11) in the GA group and 57.6% (*n* = 19) in the control group; the GA group had a significantly lower occurrence (*P* = 0.038). The frequencies of occurrence for each grade are as follows. In the GA group, 41.1% (*n* = 14), 29.4% (*n* = 10), and 2.9% (n = 1) of the patients showed oral mucositis of grades 1, 2, and 3, respectively. The corresponding values in the control group were 33.3% (n = 11), 48.5% (*n* = 16), and 9.1% (*n* = 3).

### Severity of oral pain

**Fig. 2:**
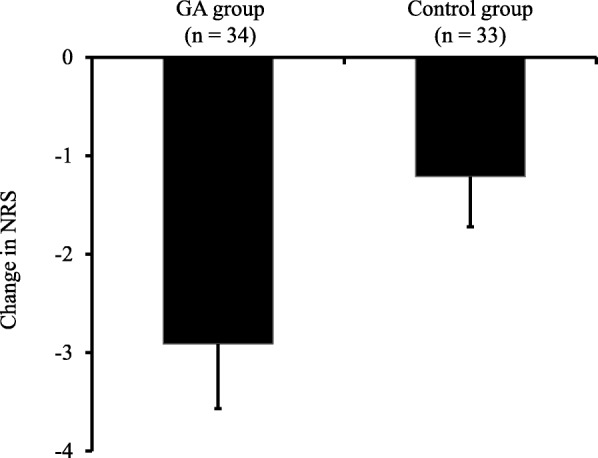
Change in NRS scores for oral pain. Changes in NRS scores were determined by comparing the NRS scores after the trial began with the NRS scores before the trial. Changes in NRS scores showed negative value in both groups, and were significantly larger in the GA group than in the control group (*P* = 0.046, Student’s *t*-test). Values are shown as mean ± standard deviation

**Fig. 3:**
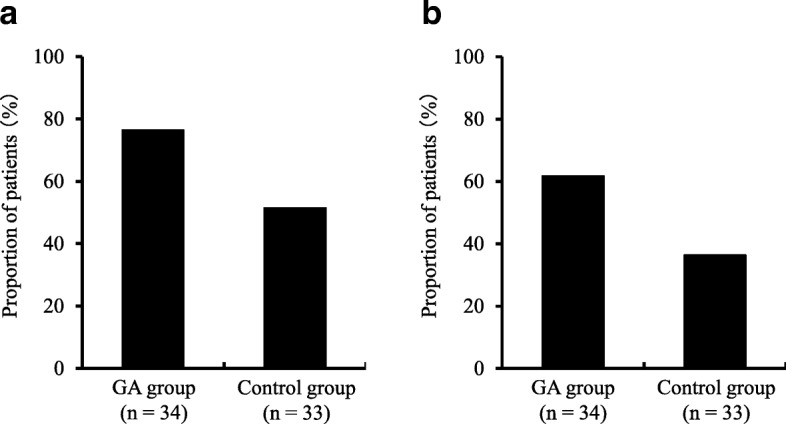
Changes in NRS scores: (**a**) clinically meaningful (≥30%) and (**b**) highly meaningful (≥50%). **a** clinically meaningful (≥30%) and (**b**) highly meaningful (≥50%)were defined based on the reduction of pain intensity from baseline. The proportion of clinically meaningful (≥30%) was significantly higher in the GA group than in the control group (**a**, *P* = 0.033 chi-square test). The proportion of highly meaningful (≥50%) was significantly higher in the GA group than in the control group (**b**, *P* = 0.038, chi-square test). This figure shows the proportion of patients (%) in whom a clinically meaningful was observed

The changes in the NRS scores for oral pain in the GA and control groups after the start of the trial are shown in Fig. 2. The change in the NRS score (mean ± standard deviation) was − 2.9 ± 0.6 in the GA group and − 1.2 ± 0.5 in the control group, with the change in the former being significantly larger than that in the latter (*P* = 0.046). The proportion of patients who exhibited clinically meaningful (≥30%) was 76.5% (*n* = 26) in the GA group and 51.5% (*n* = 16) in the control group. The proportion of patients who exhibited highly meaningful (≥50%) was 61.8% (*n* = 21) in the GA group and 36.4% (*n* = 12) in the control group. Oral pain improved more significantly in the GA group than in the control group (*P* = 0.033 and *P* = 0.038) (Fig. 3).

### Adverse events

The adverse events observed in the GA and control groups are shown in Table 2. There was no significant difference in the proportion of patients who developed haematotoxicity (neutropenia, haemoglobin decrease, or thrombopenia) between the two groups. Furthermore, the intergroup difference in the proportion of patients who showed non-haematotoxic adverse effects (nausea, vomiting, or diarrhoea) or decreased liver function (increase in aspartate transaminase or alanine transaminase levels) was not significant. One patient voluntarily stopped GA treatment due to nausea, but no other GA-related side effects were observed.

**Table 2:** Adverse events (all grades)

	GA group	Control group
	(*n* = 34)	(*n* = 33)
Hematotoxicity, *n* (%)		
Neutropenia	1 (2.9)	1 (3.0)
Hemoglobin decrease	3 (8.8)	4 (12.1)
Thrombopenia	3 (8.8)	2 (6.1)
Non-hematotoxicity, *n* (%)		
Nausea	9 (26.5)	7 (21.2)
Vomiting	1 (2.9)	0 (0.0)
Diarrhea	1 (2.9)	4 (12.1)
Fever	2 (5.9)	2 (9.1)
Decreased liver function, *n* (%)		
AST increased	1 (2.9)	1 (3.0)
ALT increased	1 (2.9)	2 (6.1)

*AST* aspartate transaminase, *ALT* alanine transaminase

## Discussion

We hypothesized that GA would prevent oral mucositis and alleviate oral pain in already affected patients among those undergoing outpatient chemotherapy regimens involving fluorinated pyrimidine or anthracycline anticancer agents.

Previous studies have demonstrated the efficacy of oral care, pain management, and nutritional supplementation for the treatment of oral mucositis occurring during chemotherapy [13, 14]. However, there is still no effective preventative measure or treatment for oral mucositis. L-glutamine has been reported to decrease the rate of occurrence of oral mucositis and severity of pain in oral mucositis among patients undergoing chemotherapy and radiation therapy [15–17]. Therefore, the efficacy of adding L-glutamine as a component of enteral nutrition to alleviate oral mucositis was investigated. However, the amount of L-glutamine added during enteral nutrition is minimal, because of which a satisfactory amount of L-glutamine cannot be absorbed. On the other hand, the advantages of GA are that it is 99% made up of L-glutamine, and it is an easy drug to take, thereby allowing an adequate amount of L-glutamine to be absorbed, and can help avoid noncompliance. In this study, we considered these advantages and used a GA suspension to be held in the mouth for 20–50 s, followed by administration of the chemotherapy drug to evaluate the efficacy of GA against oral mucositis.

The subjects in this study were patients with oral mucositis of grade 2 or higher who were undergoing a chemotherapy regimen cycle. Upon repeating the chemotherapy regimen, the chance of recurrence or aggravation of oral mucositis is high. In this patient group, oral mucositis occurred in 90% of the patients in the control group, and half of these cases were grade 2 or higher. Conversely, in the GA group, the rate of occurrence of oral mucositis significantly lowered. Furthermore, the NRS pain scores of the GA group decreased significantly more than they did in the control group. This suggests the efficacy of GA against oral mucositis due to anticancer agents.

A high GA tolerability was observed during this study in terms of safety and compliance. One patient discontinued administration of GA voluntarily, but all other patients completed their term of GA administration. Additionally, the rate of occurrence of the common side effects of GA—nausea, diarrhoea, decreased liver function—was not significantly different between the GA and control groups. This suggests that even a high dose of 9 g per day is safe for continuous administration. The occurrence of diarrhoea was slightly lower in the GA group; GA promotes the repair of intestinal mucosal tissue, which may have a protective effect against the occurrence of diarrhoea. Further, the amount of glutamine in GA did not cause any adverse effects when absorbed as part of a high protein diet [18].

In this study, a small number of patients used sodium azulene sulfonate mouthwash. Although there is insufficient evidence on the effect of sodium azulene sulfonate mouthwash on oral mucositis associated with chemotherapy, it is widely used as a prophylactic or therapeutic agent. Whether GA is superior to sodium azulene sulfonate mouthwash as a treatment strategy for oral mucositis may be an important finding, but we did not consider it in this study. Methods for the evaluation of oral mucositis was also an issue in this study. An objective evaluation of the severity of oral mucositis is difficult; therefore, we relied on a subjective method, the evaluation with which depended on the patients’ subjective symptom. NCI-CTCAE ver. 4.0, which was used in this study, is a subjective method of evaluation, which suggests that there may have been a lack of objectivity. In the future, it is necessary to conduct a more detailed investigation of the efficacy of GA by undertaking accurate oral evaluations by dentists and observing changes in symptoms over time.

## Conclusion

We have shown that GA prevents the occurrence of oral mucositis and alleviates the symptoms of oral mucositis in patients with a high risk of oral mucositis/patients undergoing chemotherapy regimens with a high risk of oral mucositis. As no countermeasures against oral mucositis have been established thus far, the results of this study may provide useful knowledge that can be used to improve the quality of life of cancer patients receiving treatment, including chemotherapy.

## Abbreviations

GA: L-glutamine
NRS: Numerical rating scale
